# Unusual Presentation of Acute Perimyocarditis Following SARS-COV-2 mRNA-1237 Moderna Vaccination

**DOI:** 10.7759/cureus.16590

**Published:** 2021-07-23

**Authors:** Fatima Khogali, Rabab Abdelrahman

**Affiliations:** 1 Medical Research Center, Hamad Medical Corporation, Doha, QAT; 2 Emergency Medicine, Hamad Medical Corporation, Doha, QAT

**Keywords:** covid-19, sars-cov-2, perimyocarditis, myocarditis, mrna-1237, moderna vaccine, cdc

## Abstract

Since the start of the pandemic, to date, around 180 million cases have been diagnosed with COVID-19 worldwide with an estimated 3.9 million death toll. Mass vaccination has taken place to control spread of infection with the most commonly used vaccines being Pfizer-BioNTech and Moderna. However, the adverse events associated with vaccination have not been fully investigated. Of concern are some serious cardiovascular events such as myocarditis, pericarditis or perimyocarditis development post-vaccination. In this report, we present an unusual case of acute perimyocarditis and pericardial effusion 10 days following the second dose of Moderna COVID-19 vaccination in Qatar. At the time of presentation, the patient presented with non-specific symptoms of headache, diarrhea, vomiting, lethargy and dehydration. COVID-19 polymerase chain reaction (PCR) was negative. Once admitted to the emergency department, she started to deteriorate with very low blood pressure readings reaching 40/33 mmHg which was treated with aggressive fluid resuscitation. After 5.5 liters of intravenous fluids, echocardiography and electrocardiogram (ECG) were performed. Findings were consistent with pericardial effusion, signs of impending cardiac tamponade and acute perimyocarditis. Cardiac biomarkers including troponin T and pro-brain natriuretic peptide (BNP) were elevated. Hospital course was complicated with cardiac arrest, acute kidney injury, disseminated intravascular coagulation (DIC) and hemodynamic instability. Eventually, the patient recovered after a three-week hospital stay and was discharged on non-steroidal anti-inflammatory medication (NSAIDs). This case report highlights the hospital course and outcome linking the second dose of Moderna vaccination and the development of perimyocarditis.

## Introduction

According to the World Health Organization (WHO), Coronavirus-19 (COVID-19) pandemic has led to the death of around 3.9 million people and affected many, prompting the need for vaccine development to lessen the complications and morbidity associated with COVID-19 [1]. Myocarditis/perimyocarditis is a rare but serious adverse event that can be reported post-vaccination. Recently, there has been an increase in reported cases of myocarditis/perimyocarditis post-mRNA second dose vaccination, especially in relation to Pfizer-BioNTech [2-7]. These cases pose a new cardiac illness link following vaccination, occurring predominantly in males below the age of 30 [8-11]. Not much literature has been reported on the development of myocarditis/perimyocarditis post-Moderna vaccination [2-4]. The reason for this development is unknown; however it can possibly be attributed to more systemic and immunologic reactogenicity of mRNA vaccines as compared to other vaccines [2,6,12]. On June 24, 2021 the CDC has declared myocarditis and pericarditis as side effects of the COVID-19 vaccines. Published literature has shown that the only other vaccine with a strong link to the development of acute perimyocarditis is smallpox vaccine [13]. Additionally, there have been 789 cases of myocarditis/pericarditis reported to the Food and Drug Administration (FDA) vaccine advisory group confirmed across all age groups following vaccination [8]. Earlier reports of this cardiac illness post-COVID-19 vaccination link were initially reported on by Marshall et. al and colleagues in April-May 2021, where seven adolescent patients developed chest pain within four days of Pfizer-BioNTech vaccination and confirmed to have acute myocarditis or perimyocarditis [2,3].

## Case presentation

**Figure 1:**
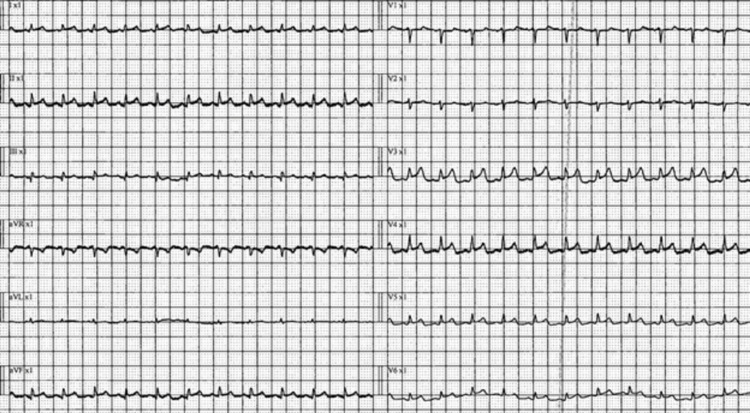
ECG showing sinus tachycardia, short PR interval, T wave abnormality and diffuse ST elevation across leads.

A previously healthy 29-year-old female with a background of food allergy to tree nuts and peanuts and partial right nephrectomy with stable mild chronic kidney disease (CKD) since birth (Urea: 8 mmol/L, Creatinine: 110 umol/L) presented to the emergency department initially on the 10th of May with fever and fatigue. Patient had no known COVID-19 exposures, history of recent viral illness or other known risk factors. His COVID-19 PCR was negative. The only positive history element was that the patient had taken the second dose of the Moderna vaccine 10 days prior to visiting the emergency. On the day of vaccination, the patient suffered from a high-grade fever measured at 38.5 degrees Celsius and continued to have low-grade fever for two days. She also complained of fatigue, myalgia and headache. Patient returned on the 12th of May with new non-specific symptoms of nausea, vomiting and diarrhea of one day duration. The patient was suspected of having acute severe gastroenteritis with dehydration and hypotension as blood pressure on admission was 80/66 mmHg. Her blood pressure kept dropping despite aggressive fluid resuscitation, so high dose inotropes and stress steroids were started to maintain adequate mean arterial pressure (MAP). ECG was consistent with pericarditis showing diffuse ST elevation and short PR interval (Figure 1).

Signs of severe ventricular dysfunction were present on echocardiography with decreases in right and left ventricular function, ejection fraction (EF) of 27%, a moderate increase in pericardial effusion and signs of impending cardiac tamponade (Videos 1,2,3).

**Video 1:** Echocardiogram showing pericardial effusion and reduced biventricular contractility (RV and LV). *RV: Right ventricle, LV: Left ventricle

**Video 2:** Echocardiogram showing pericardial effusion compressing both RV and RA with signs of impending cardiac tamponade. *RV: Right ventricle, RA: Right atrium

**Video 3:** Echocardiogram showing cardiac tamponade.

The patient was transferred to the ICU due to hemodynamic instability and the presence of combined hypovolemic, obstructive and cardiogenic shock. Dobutamine was added to improve ventricular function. However, it exacerbated the condition, causing vasodilatation. Sustained low-efficiency dialysis (SLED) was also started as the patient was anuric with acute kidney injury (AKI) and severe metabolic acidosis. However, it was not tolerated and there was pulseless electrical activity (PEA) for two minutes. There was return of spontaneous circulation (ROSC) with CPR and the patient regained consciousness. Volume expansion was achieved with further fluid boluses to overcome intra-pericardial pressure (IPP). Pericardiocentesis was done to relieve intracardiac pressure and 300 mL of fluid was drained. There was immediate improvement in cardiac indices with stabilization of hemodynamics. Continuous renal replacement therapy (CRRT) was given for ongoing acute kidney injury during the hospital course.

Extensive laboratory work-up showed markedly elevated cardiac biomarkers. Troponin T increased from 98 ng/L reaching up to 1,632 ng/L and pro-BNP increased from 2,862 pg/mL to 36,064 pg/L. Other labs including CBC, inflammatory markers, autoimmune profile, viral panel, blood, urine and stool cultures were unremarkable. Later on, multi-organ failure developed as evidenced by acute kidney injury, deranged liver function and DIC (Table 1).

**Table 1:** Significant findings during hospital course *Respiratory Panel includes: Coronavirus HKU, Coronavirus OC43, Coronavirus 229E, Coronavirus NL63, Influenza A, Influenza B, Metapneumovirus, MERS Coronavirus, Hepatitis C virus, Parainfluenza 1, Parainfluenza 2, Parainfluenza 3, Parainfluenza 4, Respiratory Syncytial Virus, Bordetella pertussis, Chlamydophila pneumonia, Myocoplasma pneumonia. *Autoimmune screen includes: anti-nuclear antibody, anti-double stranded DNA antibody, anti-ribonucleotide antibody, anti Jo-1 antibody, anti-topoisomerase I protein (Scl-70), anti-RO, anti-LA antibodies, anti-glycoprotein antibody, IgM, IgG, anti-centromere antibody (CENP), anti-glomerular basement membrane antibody (GBM)

Table 1: Significant Findings during Hospital Course			
Parameters	Initial values	Repeated values (lowest or highest)	Range
Peripheral white blood count	5.8 x10^3/uL	19.1 x10^3/uL (highest)	4.0 – 10.0
Absolute neutrophil count	3.0 x10^3/uL	13.2 x10^3/uL (highest)	2.0 – 7.0
Hemoglobin	12.7 gm/dL	7.6 gm/dL (lowest)	12.0 – 15.0
Platelet	211 x10^3/uL	51 x10^3/uL (lowest)	150-400
Alanine Transaminase	16 U/L	2,003 U/L (highest)	0-33
Aspartate Transaminase	30 U/L	2,369 U/L (highest)	0-32
C-Reactive Protein	<2 mg/L	53.7 mg/L (highest)	0 – 5
Troponin T	98 ng/L	1,632 ng/L (highest)	3 – 10
NT-Pro-Brain Natrieutic peptide	2,862 pg/mL	36,064 pg/mL (highest)	<125 pg/mL
Prothrombin Time (in seconds)	12.1 seconds	24.8 seconds (highest)	9.7 - 11.8
International Normalized Ratio	1.1	2.3 (highest)	<1.1
D-Dimer	3.34 mg/L	35.20 mg/L (highest)	0.00-0.46
Fibrinogen	1.97 gm/L	4.36 gm/L (highest)	1.70-4.20
Partial Thromboplastin time (in seconds)	28.0 seconds	55.1 seconds (highest)	24.6-31.2
Urea	11.9 mmol/L	17.9 mmol/L (highest)	2.5-7.8
Creatinine	135 umol/L	284 umol/L (highest)	44-80
Sodium	137 mmol/L	-	133-146
Potassium	3.9 mmol/L	5.6 mmol/L (highest)	3.5-5.3
Magnesium	-	0.39 mmol/L (lowest)	0.70 – 1.00
Bicarbonate	25 mmol/L	5 mmol/L (lowest)	22 – 29
pH	7.404	7.070 (lowest)	7.350-7.450
Partial pressure of oxygen	148 mmHg	313 mmHg (highest)	83-108
Partial pressure of carbon dioxide	14 mmHg	15 mmHg (lowest)	35-45
Potassium	5.4 mmol/L	5.4 mmol/L (highest)	3.5-5.0
Lactic acid	3.90 mmol/L	10.70 mmol/L (highest)	0.36-1.60
Bicarbonate	8.6 mmol/L	4.7 mmol/L (lowest)	23.0-29.0
Pericardial fluid hematology	Color: yellow, slightly turbid WBC: 39 (showing mixed neutrophils, lymphocytes and monocytes)	-	-
Viral Panel			
COVID-19 PCR	Negative	-	-
Cytomegalovirus PCR	Negative	-	-
Adenovirus PCR	Negative	-	-
Epstein-Barr virus PCR	Negative	-	-
Rhino/Enterovirus PCR	Negative	-	-
Autoimmune Profile			
Rheumatoid Factor	Negative	-	-
Anti-neutrophilic cytoplasmic antibody	Negative	-	-
Anti Cardiolipin antibody IgG	Negative	-	-
Anti Cardiolipin antibody IgM	Negative	-	-
Lupus anticoagulant antibody	Negative	-	-

Although the patient had a complicated hospital course, there was drastic recovery over a three-week period. She stabilized hemodynamically, weaned off inotropes and all her labs improved including cardiac biomarkers. Serial ECG’s showed no significant changes and echocardiography showed minimal pericardial effusion post-pericardiocentesis. She was diagnosed as a case of perimyocarditis and discharged on colchicine and aspirin to be followed in the cardiology outpatient clinic in four to six weeks.

## Discussion

Perimyocarditis is used when a patient has evidence of both pericardial inflammation and myocardial abnormalities with elevated cardiac enzymes. Clinical presentation varies from mild symptoms such as chest pain, fatigue, shortness of breath to severe presentations such as arrhythmias, syncope or even cardiac arrest. Etiology of perimyocarditis includes infectious and non-infectious etiologies related to autoimmune, toxin/drug or radiation exposure and vaccine-related perimyocarditis. The diagnosis of perimyocarditis relies on clinical history and findings with supportive evidence from cardiac biomarkers and imaging including echocardiography and cardiac MRI [14,15].

The true baseline of myocarditis and pericarditis is rare occurring at 1 to 10 cases per 100,000 persons and at 0.1%, respectively. Currently reported literature has concluded on the probability link between the second vaccine dose and the onset of myocarditis, especially observed in males aged 16 to 24 years [2-5, 8-10,12,14]. The majority of these cases occurred in the United States and was related to Pfizer-BioNTech. The reported incidence by the CDC estimates this effect to be around 13 cases per 1 million of administered doses in the U.S [16-18]. In Canada, 25 cases of myocarditis/pericarditis have been confirmed by the Public Health Agency of Canada (PHAC), and in Israel, 275 cases were confirmed by the ministry of health, predominantly in young males, mirroring the reports in the U.S [5,9].

This side effect is said to be rare and the possibility of developing a severe reaction post-COVID-19 vaccination is unlikely [18]. However, in our reported case, our patient developed a complicated hospital course which was associated with a high morbidity. This was due to the presence multiple factors such as cardiogenic shock in the form of biventricular failure, cardiac arrest, acute kidney injury and DIC. A thorough work up for labs and imaging were negative and inconclusive towards a diagnostic cause of perimyocarditis, except for recent COVID-19 vaccination history.

The events complicating our case hold a unique but significant importance to vaccine-related adverse effects of the Moderna vaccine. The rapid accumulation of pericardial fluid leading to cardiac tamponade followed by cardiac arrest in an otherwise healthy young patient is worth further investigations as vaccine immunogenicity is not fully understood. Other complications such as severe acute kidney injury requiring dialysis or deranged liver function requiring fresh frozen plasma may raise our suspicion to other possible side effects of the vaccine other than perimyocarditis [16,17]. Furthermore, most of the cases reported by the CDC had an uncomplicated hospital outcome and recovered fully unlike our patient who became very sick. This points to a possibility of increased reactogenicity in some patients due to COVID-19 vaccination [16-18]. In addition to this, the true incidence of myocarditis and perimyocarditis is currently unknown in Qatar and it is important to establish as the rate may be higher as compared to the U.S due to a smaller population average of 2.8 million people [19].

However, it is important to note that we are limited in our observations as these sequelae could have been contributed by hypoperfusion in the case of acute kidney injury or derangement of liver function as a consequence of post-cardiac arrest. Further research is required in our unique case to understand the association between vaccination and perimyocarditis development and other side effects.

## Conclusions

Despite the rising need for the COVID-19 vaccines worldwide, further investigations are required taking into consideration the recent reported literature on possible side effects. Perimyocarditis, in particular, might be the current interest with very recent literature reported by the CDC.

It is also crucial that as physicians we keep an open mind when diagnosing patients. Our 29-year old patient presented with atypical symptoms pointing to an unlikely diagnosis of perimyocarditis. The presence of atypical or vague symptoms post-COVID-19 vaccination should raise the suspicion for possible adverse events. We recommend thorough investigations for patients presenting to the emergency, such as CBC, renal function test, liver function test, ECG, cardiac biomarkers and echocardiography, so as not to miss any important diagnoses. Lastly, we also recommend more research to be done on this topic, specifically to establish the incidence rate between the Moderna vaccination to perimyocarditis and the long-term effects associated with this link.
